# Telehealth in remote Australia: a supplementary tool or an alternative model of care replacing face-to-face consultations?

**DOI:** 10.1186/s12913-023-09265-2

**Published:** 2023-04-05

**Authors:** Supriya Mathew, Michelle S. Fitts, Zania Liddle, Lisa Bourke, Narelle Campbell, Lorna Murakami-Gold, Deborah J Russell, John S. Humphreys, Edward Mullholand, Yuejen Zhao, Michael P. Jones, John Boffa, Mark Ramjan, Annie Tangey, Rosalie Schultz, John Wakerman

**Affiliations:** 1 Menzies School of Health Research, Charles Darwin University, Alice Springs, NT Australia; 2 Institute for Culture and Society, Western Sydney University, Parramatta, NSW Australia; 3 Department of Rural Health, The University of Melbourne, Shepparton, VIC Australia; 4 Flinders Rural and Remote Health Northern Territory, College of Medicine and Public Health, Flinders University, Darwin, NT Australia; 5 PocheSA+NT, Flinders University, Alice Springs, NT Australia; 6 School of Rural Health, Monash University, Bendigo, VIC Australia; 7 Miwatj Health Aboriginal Corporation, Nhulunbuy, NT Australia; 8 Northern Territory Department of Health, Darwin, NT Australia; 9 School of Psychological Sciences, Macquarie University, North Ryde, NSW Australia; 10 Central Australian Aboriginal Congress, Alice Springs, NT Australia; 11 Top End Population and Primary Health Care, Northern Territory Government, Casuarina, NT Australia; 12 Ngaanyatjarra Health Service, Alice Springs, NT Australia

**Keywords:** First Nations, Aboriginal people, Telemedicine, Video consultation, Remote consultation, Digital health, Telehealth

## Abstract

**Background:**

The COVID-19 pandemic increased the use of telehealth consultations by telephone and video around the world. While telehealth can improve access to primary health care, there are significant gaps in our understanding about how, when and to what extent telehealth should be used. This paper explores the perspectives of health care staff on the key elements relating to the effective use of telehealth for patients living in remote Australia.

**Methods:**

Between February 2020 and October 2021, interviews and discussion groups were conducted with 248 clinic staff from 20 different remote communities across northern Australia. Interview coding followed an inductive approach. Thematic analysis was used to group codes into common themes.

**Results:**

Reduced need to travel for telehealth consultations was perceived to benefit both health providers and patients. Telehealth functioned best when there was a pre-established relationship between the patient and the health care provider and with patients who had good knowledge of their personal health, spoke English and had access to and familiarity with digital technology. On the other hand, telehealth was thought to be resource intensive, increasing remote clinic staff workload as most patients needed clinic staff to facilitate the telehealth session and complete background administrative work to support the consultation and an interpreter for translation services. Clinic staff universally emphasised that telehealth is a useful supplementary tool, and not a stand-alone service model replacing face-to-face interactions.

**Conclusion:**

Telehealth has the potential to improve access to healthcare in remote areas if complemented with adequate face-to-face services. Careful workforce planning is required while introducing telehealth into clinics that already face high staff shortages. Digital infrastructure with reliable internet connections with sufficient speed and latency need to be available at affordable prices in remote communities to make full use of telehealth consultations. Training and employment of local Aboriginal staff as digital navigators could ensure a culturally safe clinical environment for telehealth consultations and promote the effective use of telehealth services among community members.

## Introduction

Residents of rural and remote areas of Australia, comprising more than a quarter of the total population, face poorer access to health care services and significant inequities in health outcomes compared to their city counterparts [1, 2]. Rural and remote areas are home to a large proportion of Australian Aboriginal and Torres Strait Islander peoples (hereafter referred to as Aboriginal), who speak a range of languages and who have strong cultural knowledge and ties, and also experience a burden of disease that is more than double the rate of non-Aboriginal Australians [3]. The high burden of disease coupled with low access to primary health care (PHC) and health professional shortages in rural and remote locations [4] exacerbates the poor health outcomes of Aboriginal people. Telehealth has been proposed as a key strategy to improve access to healthcare and overcome shortages of health staff in remote areas [2, 4].

The COVID-19 pandemic has accelerated the use of telehealth (defined in this paper as the use of telephone or video calls for medical consultations) in PHC in Australia and resulted in telehealth becoming more normalised as a means of delivering PHC. In 2020, the Australian Government made temporary changes to the Medicare Benefits Schedule (MBS) to enable subsidised access to PHC services that were provided via telephone or videoconferencing by General Practitioners (GPs), medical practitioners, specialists, consultant physicians, nurse practitioners, participating midwives, allied health providers and dental practitioners conducting oral and maxillofacial surgery. In the same year, additional incentives were also provided to GPs and other health practitioners to ensure continued access to essential health care services for all Australians [5]. Some of the MBS arrangements for telehealth consultations (telephone and video) for patients living in remote communities continue (as of 29th November 2022), making it particularly important to understand the implications for their increased use in remote communities.

Despite the voluminous literature on telehealth, significant gaps in knowledge still exist about when and how to use telehealth effectively in remote PHC services and with Aboriginal people living in remote settings. One pre-pandemic systematic review (2017) of Australian telehealth services identified some of the benefits of telehealth to include - a reduced need for travel, greater access to specialist services, lower costs compared to face to face consultations, improved clinical outcomes (decrease in prevalence rates of ear diseases), decreased missed appointment rates, increased screening rates and better social and emotional well-being that was attributed to the ability to receive care in community, stay with family while receiving care and the potential choice available for palliative patients to die in Country [6]. This review primarily included studies that discussed telehealth consultations with non-GP medical specialists and allied health professionals (e.g. psychiatrists, ophthalmologists, palliative care physicians, oncologists, speech and language therapists, ear nose and throat specialists and anaesthetists). Another review of rural and remote Australian telehealth services also found no studies discussing telehealth consultations between GPs and their patients [7]. A review of international literature suggests that while Aboriginal people are mostly satisfied with telehealth for chronic disease management, its acceptability among Aboriginal people is dependent on culturally safe telehealth service delivery [8]. In short, there is a dearth of evidence on the use of telehealth consultations for PHC consultations in remote Aboriginal communities in Australia.

Against this background, it is critical that the experiences and preferences of both health staff and consumers in remote communities in Australia in using telehealth are documented and key elements that support and limit the effective use of telehealth in this context are well understood. This paper examines the perspectives of staff working mainly in remote PHC clinics in regard to the use, drivers, and limitations of conducting specialist and PHC consultations via telehealth.

## Data and methods

### Setting

**Fig. 1:**
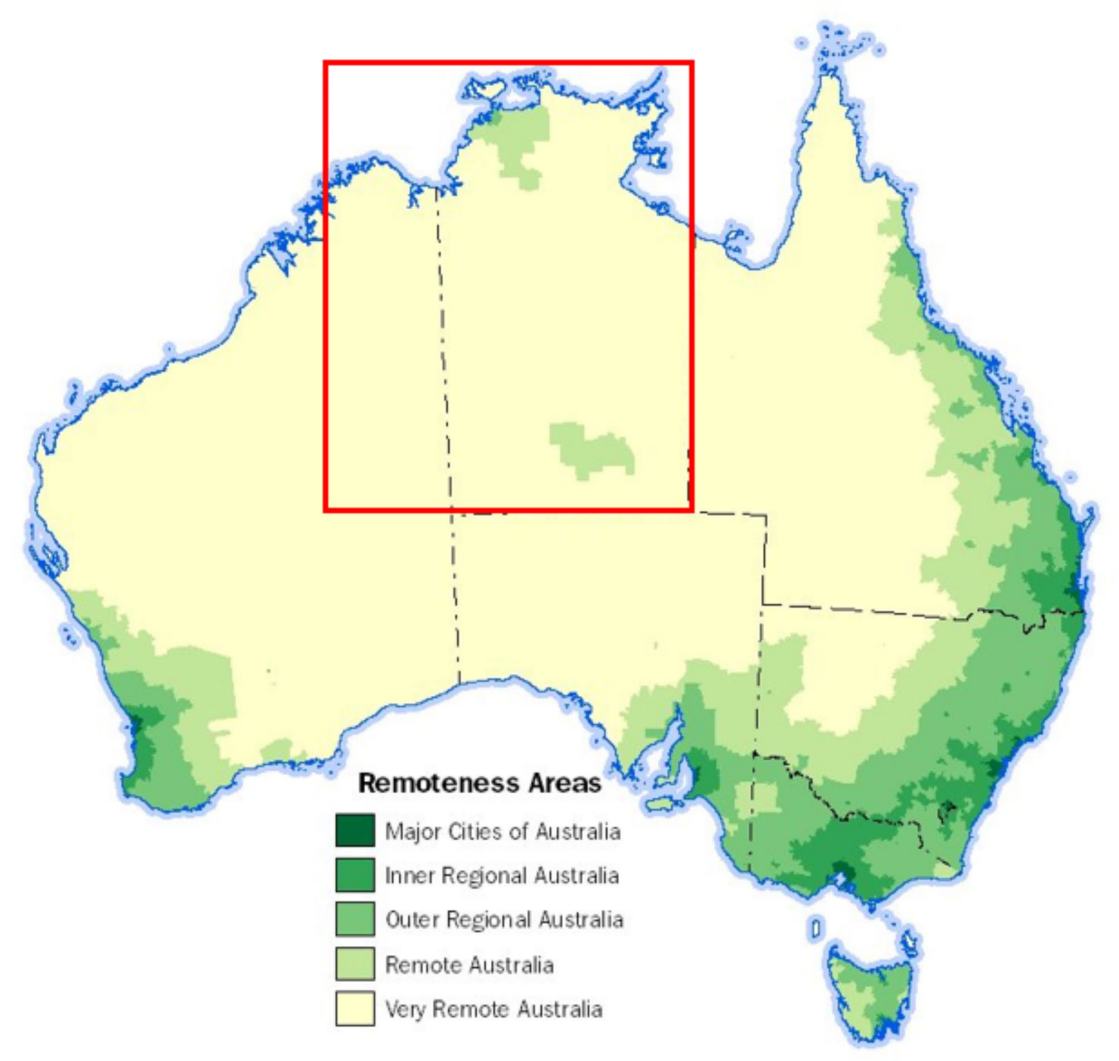
Remoteness Structure in Australia. The red rectangle shows the study area Source: ABS Australian Statistical Geography Standard (ASGS) Volume 5 – Remoteness Structure (cat. no. 1270.0.55.005), Remoteness Structure (abs.gov.au).

This study is a part of a larger three-year mixed-methods study exploring the impact of short-term staffing on Aboriginal Community Controlled Health Services (ACCHSs), staff and clinic users and investigating how the COVID-19 pandemic has affected the provision of PHC services [9]. Participants were recruited from eleven ACCHSs, which are Australian PHC services governed by local Aboriginal communities with the aim of delivering holistic, comprehensive, and culturally appropriate health care [10]. The participating ACCHSs were from the Northern Territory (NT) and Western Australia (WA) (see Fig. 1). All but one of the twenty communities serviced by the participating ACCHSs were classified as remote or very remote by the Australian Bureau of Statistics (ABS) [11]. Access to the remote communities varied, with some via sealed roads, and others with only unsealed roads that are often impassable during the wet season in northern Australia.

### Participants

Staff from the eleven ACCHSs were invited to be interviewed. Participants had varying levels of experience working in the clinics and included PHC clinic staff (drivers, Aboriginal Health Practitioners (AHPs), Remote Area Nurses (RANS), GPs, other health workers such as Aboriginal Liaison Officers, a range of administrative staff such as Human Resources (HR) personnel, clinic managers and customer service officers), medical retrieval staff and leadership staff from both individual ACCHSs and the peak bodies representing the community-controlled health sector.

### Participant recruitment and data collection

Data collection commenced in February 2020, just before the Australia-wide COVID-19 ‘lock down’ when interstate and international travel were severely restricted. Field work recommenced in June 2020 and finished in October 2021. During this period, a range of policy announcements (such as funding for telehealth MBS items, introduction of travel and quarantine restrictions, and biosecurity zone declarations for remote Aboriginal communities) were made by various levels of government and non-government organisations [12, 13]. These policies were designed to restrict the spread of COVID-19, but ensure that remote communities had access to essential services including PHC services.

As most ACCHSs deliver PHC to multiple communities, senior representatives of participating ACCHSs who comprised the project Steering Committee provided guidance on which communities and community clinics to visit. Prior to each visit, the research team liaised with the local clinic manager, who then disseminated project information to all clinic staff. Whenever possible, two members of the research team attended staff meetings to explain the project.

A topic guide was created for the semi-structured interviews by the research team, which was informed by a similar, previous study exploring the impact of short-term staffing in remote clinics run by the Northern Territory Government (NTG) [14, 15]. COVID-19 specific questions covered several topics including: (i) workforce and resources challenges; (ii) responses by the local clinic and ACCHS to address the challenges; and (iii) lessons learnt. Telehealth, the main focus of this paper, emerged as a key COVID-19 response strategy used by ACCHSs to ensure continued health service delivery for remote communities during the COVID-19 period.

### Data analysis

Audio-recordings were transcribed by a professional transcriber and then checked for accuracy against the original recordings. Each interview was assigned a unique identifier. Three authors conducted a thematic analysis of the data taking an inductive approach. NVivo v12 software (QSR international) was used. Six initial interviews with participants who had different roles were read by two members of the research team who independently created initial codes. One author then coded all remaining transcripts to identify patterns in the codes and links between the codes thereby organising the data into meaningful themes [16]. To validate the emergent themes, three further interviews from each of the ACCHSs were independently coded by two of the research team members. Any disparities identified were discussed and resolved.

### Ethics

The study had ethics approval from the Human Research Ethics Committee of the NT Department of Health and Menzies School of Health Research (project number DR03171), Central Australian Human Research Ethics Committee (CA-19-3493) and Western Australian Aboriginal Health Ethics Committee (WAAHEC-938).

## Results

Data were collected from 248 staff working in the clinics run by the participating ACCHSs. One-third of the staff members interviewed identified as Aboriginal. Approximately 60% of the participants were clinicians and were directly involved in telehealth consultations. The remaining non-clinician participants were able to comment on the use of telehealth based on their non-clinical interactions with patients or organisational knowledge. Aboriginal staff were able to comment on their personal experiences and feedback they received from family and friends. Overall, telehealth was perceived by staff members to be an important strategy used by ACCHSs to ensure that clinic users could consult their regular GP, allied health practitioners and specialists during the COVID-19 lock down period.

Four themes emerged during the coding process: (1) contexts in which telehealth works well and not so well; (2) additional resources required for effective telehealth consultations; (3) opportunities available through the utilisation of telehealth; and (4) telehealth: a supplementary tool or a replacement model?

### Contexts in which telehealth works well and not so well

In the initial months of the pandemic, staff indicated that many community members chose not to visit the clinic in person to meet with clinic staff face-to-face due to their concerns about contracting COVID-19. Telehealth – especially the MBS funding of telephone consultations – enabled PHC staff the flexibility of offering virtual PHC services which averted the need for community members to attend the clinic in person. However, staff acknowledged that the utility of telehealth consultations from the PHC clinic to community members in their homes was limited by community members not consistently having private access to working telephones.

*“[COVID] just brought a lot of fear when it first came in, [community members were] very reluctant to come in. We did offer telehealth to some of our clients that have working phones, which we still offer” (ACCHS 6).*

Patient access to smartphones that had videoconferencing technologies installed (for example Skype, Microsoft Teams) was discussed as a critical feature for more effective telehealth consultations. Many clinic users had *“phones, but it’s not equipped, like it’s not really smartphones” (ACCHS 1).* Access to a working phone was also problematic as: *“majority of the time the phones were disconnected” (ACCHS 1)* and thus staff found it difficult to contact patients in a timely way.

Staff reported that telehealth worked well when clients had good health literacy, including knowledge about their personal health conditions. A staff member highlighted this by quoting her personal experience managing her chronic disease via telehealth:

*“I handle it [telehealth consultation] okay because I know a fair bit about my condition but [not sure] whether that non-personal connection works on the Aboriginal population[sic]” (ACCHS 2).*

Staff observed that Aboriginal patients are often not *“comfortable talking to screens” (ACCHS 3)* and that audio quality during telehealth consultations is frequently suboptimal in remote communities. This perception was consistent with other bandwidth challenges staff identified, for example with clinics and community members not having the fast internet connections needed for high quality video consultations and frequent internet outages. Some of the remote communities and clinics relied on satellite rather than broadband digital connectivity, which didn’t work as well on overcast days:

*“The only problem is if it’s too cloudy and not much of visibility, the satellite [internet speed] runs really slow, like a snail. So, what we do is we turn on the telephone … and even though you couldn’t see much of the scratchy picture of the monitor, you can still understand what the doctor wants you to do” (ACCHS 4).*

Across remote northern Australia, where this study was situated, Aboriginal people predominantly speak Aboriginal languages as their first language. Language barriers between health care providers and patients were considered by staff to affect the quality of telehealth consultations, with primary health care staff prioritising telehealth consultations for community members who spoke English: “*if you spoke English it was really a no brainer, telehealth was fine, and so those clinics [consultations] worked really well” (ACCHS 3).* Staff reported making decisions about which clients to prioritise for telehealth consultations and avoiding telehealth for those where ‘*the consult would be too nuanced and [the doctor] wouldn’t be able to pick it up with the language barriers (ACCHS 3).*

Staff perceived that consultations were more acceptable to patients when the community member had an existing relationship with their health care provider. If that was the case, the telehealth consultations could be almost as effective as face-face to consultations, as explained by a diabetes educator:

“*Telehealth is great for me as a Diabetes Educator. If you already have those relationships [with your patients] in place. So, I had a core group of people that I’ve known for years and they were happy to talk*” *(ACCHS 5).*

### Additional resources required for telehealth consultations

One of the problems highlighted by staff mainly working in short-staffed clinics was the need for additional staff to support patients’ telehealth appointments. Staff reported the need for “*a clinician off the floor to sit with a patient during a telehealth consult” (ACCHS 11).* The downside of community-based staff facilitating telehealth consultations was that telehealth consults could become a dialogue between two clinicians rather than between a patient and their doctor: “*Patients often withdrew from the process and the experience, and it just became two clinicians talking to each other, which was not really what it [the consultation] should be about” (ACCHS 10).* A language interpreter was usually required to support communication between the health professional and the patient. Trained interpreters were not, however, always available.

Staff highlighted the need for interpreters during telehealth consultations, while acknowledging that interpreters were also often needed for face-to-face consultations. For some telehealth consultations, it was difficult for interpreters to translate the medical terminology adequately to patients. Staff mentioned that an interpreter *“can’t just be an interpreter that just understands the language. They really have to understand medical terms. It’s quite a specialised field, to be able to explain disease processes, or medication, or procedures that the person might have to have in hospital” (ACCHS 3).* To help explain the information to the patient, each telehealth consultation required a remote clinic staff member and an interpreter to be present with the patient during the telehealth consultation. Often family members or AHPs served as interpreters, but some staff thought the use of AHPs as interpreters “*devalues their role as a clinician” (ACCHS 11).*

Telehealth consultations also generated additional administrative work for staff. For example, the NTG and the community-controlled health services use different medical records software. This meant specialists, who are mostly employed by NTG, could only directly access patient records held by the ACCHSs during face-to-face consultations at the ACCHS. However, during telehealth consults, the local ACCHS staff described “*quickly printing and scanning documents [and sending to the specialist who then had to] check their emails for the documents” (ACCHS 3).*

### Opportunities available through the utilisation of telehealth

With the onset of the COVID-19 pandemic, telehealth became more normalised for everyday primary healthcare practice: *“more patients have been involved in telehealth [since the pandemic]” (ACCHS 11).* There were several benefits to telehealth that staff described for both PHC and specialist consults during the COVID period. Many specialists and PHC staff were located interstate when lockdowns occurred and thus several clinicians began using telehealth, which eliminated their previously long travel times to remote communities. Clinicians recollected that they “*used to spend a lot of time travelling between communities which eats up a lot of [their clinical consultation] time [as] a GP” (ACCHS 4).* In some instances, this saved travel time was reallocated to health service delivery:

*“Staff were able to get a lot more people with GP management plans and allowing doctors to work off-site and follow up with those patients as they needed, and more renal telehealth visit consults via the phone, and do group sessions where they’ll try to get 10 people in a day and they’re all done by the telehealth systems as well” (ACCHS 5)*.

*“…we’ve got a GP who’s a point seven [0.7 Full Time Equivalent], who comes from South Australia and he was coming up to [name of the urban centre] regularly, and when COVID struck and he couldn’t get across the border…so when Medicare introduced the telehealth as a Medicare item, when that was passed, he was able to work from [name of the urban centre]… and, [he] works down there now and the nurses find it really good because they can pick up [the telephone and consult with the doctor]” (ACCHS 5)*.

Some clinics had regular GPs who were categorised as more vulnerable to COVID (due to age or pre-existing medical conditions). The introduction of a telehealth option meant that such vulnerable staff could safely continue delivering PHC services with their regular clients, despite being based in a different location.

*“One of our doctors here is doing telehealth COVID stuff…..today she’s doing COVID calls and she’s also over 55, so it’s probably better for her to do a role like that, than be exposed [to the virus]” (ACCHS 1).*

Telehealth also provided an alternative to patients having to travel to regional centres to access specialist appointments. Remote clinic staff reported that some patients preferred not to travel as staying on Country (within the community) enabled them to meet family or cultural obligations locally. During COVID-19 lockdown periods processes to gain permission to travel from a remote community to a regional centre changed regularly, as did the rules for travel. Processing times could also be lengthy. Some community members therefore preferred not to travel for medical appointments as they were worried about not being allowed to return to the community. For example, there were reported instances involving unwell children:

“*Kids needed to [see a doctor at the hospital]… and mums were afraid that they wouldn’t be able to get back, because it took so long, even for the hospital to provide an exemption” (ACCHS 11).*

Another perceived advantage of telehealth consultations was that primary health care staff were able to participate in specialist appointments with their patients. As these appointments were otherwise usually based in regional centres, telehealth provided remote clinic staff the opportunity to obtain a better understanding of their patients’ medical conditions, management and treatment plans:

*“Telehealth is valuable for that you can have three [patient, specialist and clinic staff] to be together, ‘cause we [clinic staff] wouldn’t be able to go to town [for face-to-face specialist appointment]” (ACCHS 10).*

*“For example, I had a patient with a fracture, I can talk directly with that orthopaedic team and the patient and so we can all be there together for a conversation and say, okay, this is what we need to do and this is why” (ACCHS 11).*

### Telehealth: a supplementary tool or a replacement model?

This theme was frequently discussed by participants who were clinicians. Staff thought telehealth would “*continue on as a way of delivering care” (ACCHS 10)* after the COVID period, especially for specialist appointments. There was a common view among ACCHS staff that telehealth consults were a way to augment rather than replace face-to-face consultations: “*I think it [telehealth] could be a way of supplementing our [doctor] visits into the future. But I don’t think you [telehealth consultations] can beat having someone here” (ACCHS 3).* A hybrid model that utilises both telehealth and face to face health service delivery was considered to provide “*greater access to the specialists on a needs basis” (ACCHS 3)*, as often there are requirements for follow-up appointments not long after a specialist has visited the community. Staff also felt that medical specialists could use telehealth to follow up patients who were recently discharged from regional hospitals. Such appointments if planned properly with the local clinic to ensure patient is back in the community and there was adequate time for any required tests to be ordered was considered to work well by staff.

In some communities, a hybrid model approach continues for GP consultations as well, where telehealth consultations are arranged with regular GPs, whenever face-to-face consultations are unavailable: *“So now patients can access their GP via telehealth if we don’t have one available in the clinic and we’ve been able to continue with that, which is fantastic” (ACCHS 5)*.

## Discussion

**Fig. 2:**
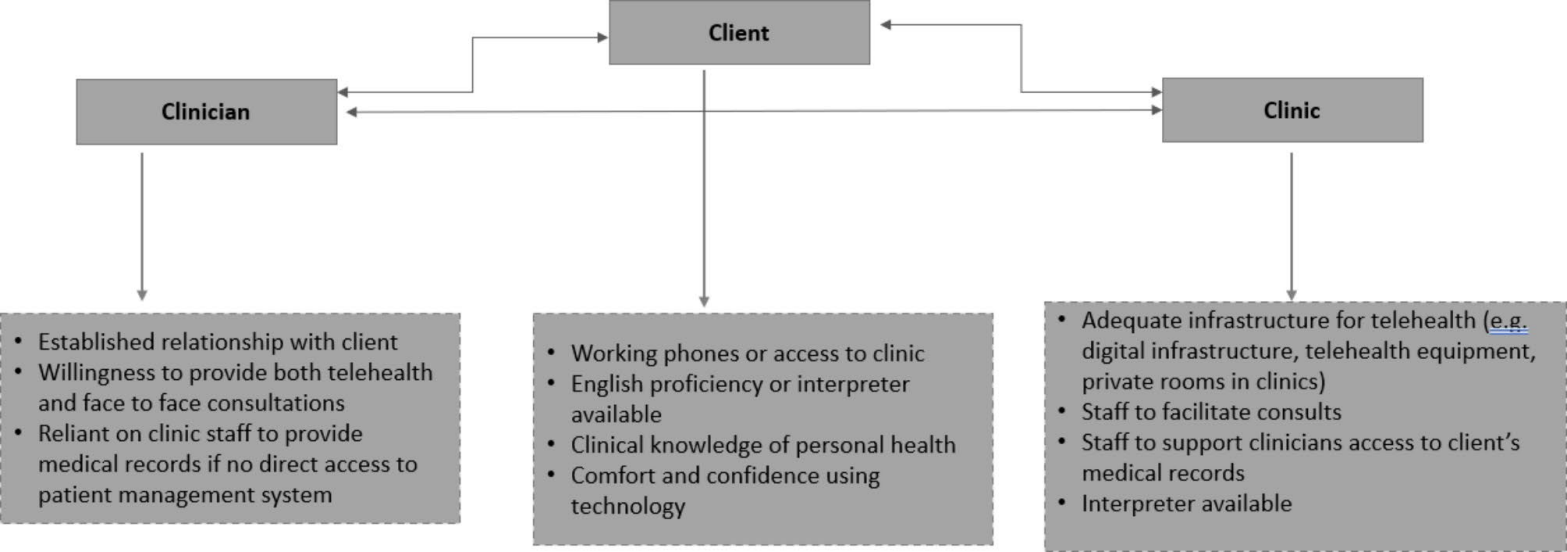
Elements that affect successful telehealth delivery

The COVID-19 pandemic was a catalyst for increased telehealth uptake internationally and locally alike [17–20]. In remote Australia, use of telehealth increased as health services sought alternatives to face-to-face care as the impact of COVID-19 travel restrictions and risk of spread of infection limited movements of patients and health professionals into and out of remote communities. Nonetheless, while health care access was enabled by implementing telehealth across remote communities in the context of the pandemic, it is important to carefully consider the pros and cons of ongoing telehealth use with remote community members. This study indicates that successful telehealth delivery for community members living in remote communities is dependent on (i) the availability of basic infrastructure required for telephone or video consultations whether facilitated by a clinic staff at a clinic or conducted at a client’s home (ii) pre-existing relationship between the patient and the health care provider and (iii) specific characteristics of clients and circumstances in which health staff operate (see Fig. 2).

Telehealth consultations in remote communities were mostly conducted at the clinic with assistance from clinic staff. This contrasts with the patient’s home as the more common location for patients living in urban settings. Occasionally remote clinic staff try to contact patients by phone, but this is often ineffective as less than half of the Aboriginal population in remote locations own a smartphone [21], and many patient phones are not operational or are disconnected due to not being recharged in a timely way. For telephone consultations with patients to occur outside of remote clinics, it would require (1) availability of phone coverage outside of the clinic that patients can use and (2) access to operational phones, which potentially may require patients to be provided with phone credits. Mobile phones are commonly shared between people in Aboriginal communities [22], which makes it challenging to contact a patient directly and also raises medical privacy issues. In summary, the patient-end (usually a remote clinic) of a telehealth consultation needs to be adequately resourced (e.g. devices suitable for telehealth set up within the remote clinic with adequate internet connections [23]) for the successful delivery of telehealth consultations. The Australian Government has been working towards ensuring that Aboriginal people have equal levels of digital inclusion by 2026, which would greatly support telehealth delivery into the future [24]. Low orbiting satellite connections promise to offer the stable, high speed, low latency connections that are needed at a reasonable cost, but this needs to be accessible in all remote communities [25].

As also depicted in Fig. 2, a pre-existing relationship between the clinician and the patient is perceived by remote clinicians as being important for a successful telehealth consultation, particularly in primary health care settings. This is consistent with the findings of a pre-COVID systematic review which found that Aboriginal Australians mostly appreciated the ability to have telehealth consultations with their specialists or regular GPs who were known to them [6]. This study also emphasises the need for hybrid models of care that offer both face-to-face and telehealth consultations, as found in other studies [20].

Client specific characteristics can affect telehealth delivery (see Fig. 2). Clinical knowledge of personal health and adequate health literacy can contribute towards the effectiveness of a good telehealth consultation. This is consistent with previous studies, citing patients’ incorrect use of prescribed medications and their inability to describe their symptoms to the doctor during telehealth consultation [17]. Patients’ English language proficiency [20, 26] and confidence using technology [27] have also been identified as important for effective telehealth consultations.

The availability of remote clinic health staff – primary health care staff and interpreters – to facilitate consultations can also affect effective telehealth delivery. Many telehealth consultations with remote clinic users require the assistance of interpreters or family members or AHPs to enable translation and to support cultural safety. There may be an emerging role for local digital health navigators who – with a basic understanding of health terminology, symptomology, and medical practices – can support language translation and cultural safety, use and troubleshoot smartphone technology and promote the uptake of telehealth through community engagement and education [28]. From a remote clinic staff point of view, telehealth consultations were considered resource intensive as staff had to ensure that patients were in the clinic at the required time, a private room was available for their use, there was a staff member available to facilitate the consultation, they were prepared for the consult (e.g. reports were printed and sent to the clinicians and any required bloods or other tests had been done), while also managing the waiting process for their patients as the doctor consulted with other patients by telehealth. Differing patient management software used by the community controlled primary health sector and by specialists introduces patient medical record access issues for doctors, contributing further to the workload of remote clinic staff. Harmonising different patient management software systems across and within jurisdictions could reduce such access issues and any administrative work related to it. As remote clinics have limited space, are frequently understaffed and have high staff turnover [4], any additional resourcing and workload requirements contribute a substantial additional burden on staff members’ heavy workloads. Continued and increased telehealth roll-out will need to carefully consider impacts on remote clinic staff and clinic operations and ensure adequate resourcing is provided. Additional administrative support could enable a lot of the required work to manage appointments to be taken off busy clinicians and this could be done by local, digital health navigators.

Despite, challenges, telehealth offers a wide range of benefits to both community members and clinicians. Increased telehealth consultations during the COVID period enabled remote clinic users to maintain contact with their regular GPs or specialists who were unable to travel to communities during the pandemic. Reduced travel time was perceived as a benefit and has frequently been reported as an advantage of telehealth consultations in other literature [23, 26, 27, 29–34]. Travel time in remote Australia is substantial due to difficult and potentially dangerous unsealed and unfenced roads, limited public transport (e.g. infrequent bush buses to transport people between regional centres and remote communities; absence of regular flights to some communities) and high expenses related to remote travel (e.g. costs of travel and accommodation) [35]. Reduced travel time for doctors meant they were able to allocate more of their time for clinical consultations, while for patients it meant fewer missed medical appointments, as patients could more easily attend a telehealth appointment and meet local family obligations and participate in prioritised cultural ceremonies [35]. Patients could have family members present in the consultation to enable shared understanding of medical advice, and were also less likely to be disconnected from their usual social/family supports as a result of travelling for specialist medical care [36], with positive effects for their mental and social well-being.

Telehealth as a supplementary model can potentially enhance access to care, including improving access for male clinic users who often prefer to see a male health professional [37]. Telehealth has the potential to also improve access for patients who are concerned about medical confidentiality, which can be problematic in small, tightly-knit communities serviced by clinics where local staff are employed [38]. Previous studies have explored the dilemmas experienced by local health care workers, particularly related to gaining community trust, ensuring local staff safety [39] and providing culturally respectful and safe care. For example, avoidance relationships followed by Aboriginal community members means certain family members are not allowed to meet face to face or speak directly with certain family members. This restricts consultations between some clinic users and local staff and in such situations a different clinician will need to provide care [40, 41]. Some of these cultural barriers can be resolved by providing telehealth as an alternate option to face-to-face consultations.

The cost effectiveness of telehealth consultations should be reviewed in light of the real, but often ‘hidden’ costs relating to additional resourcing requirements in remote communities (availability of clinic or other private consultation spaces, availability of remote clinicians, interpreters and other potentially new cadres of support staff such as digital health navigators [28] or administrative support staff, provision of functional digital devices and mobile phone credits). These should be weighed against cost savings related to patient and clinician travel to/from remote communities [42]. The overall effectiveness of telehealth consultations in remote communities should also capture less tangible costs and benefits, such as the reduced opportunity to develop trust with a patient during virtual meetings, the ability of remote community members to stay on Country (within their community) and its contribution to the overall social and emotional well-being of community members [43].

The main strength of this study is that it includes the perspectives of many remote area staff who have first-hand knowledge of the effectiveness of both PHC and specialist telehealth consultations offered to remote community patients. While the study is focussed on telehealth for remote Australian Aboriginal communities, some of the findings are applicable to other First Nations populations living in remote regions in countries such as Canada. The data were collected during the COVID-19 pandemic, so the responses undoubtedly reflect the resultant constraints, for example, community members hesitating to attend clinics and remote clinic staff not being able to visit community members in their homes during lockdowns. Another limitation of this paper is that it captures the perspectives of remote health service staff and does not directly reflect consumer perspectives. To some extent this limitation was addressed, as one third of the staff members interviewed were Aboriginal and had either friends or family members using the clinic, which meant Aboriginal staff were able to provide a broader perspective which included both patient and provider experiences.

## Conclusion

Well-resourced telehealth can improve access to health care for remote Australians, particularly when it complements face-to-face visits by doctors and other health professionals. However, in terms of equity of health care access, telehealth appointments should be a choice that is available for remote community clients and not the only option for receiving health services. Over-dependence on telehealth could exacerbate the high workloads of remote clinic staff and negatively affect care for community members. Patient end-support in the form of adequate facilities for telehealth consultations (fast internet connections) and local digital navigators who can facilitate and encourage telehealth consultations are important. It is also important to understand the real costs of telehealth consultations for remote communities before rolling it out.

## Data Availability

The qualitative data collected for this study was de-identified and aggregated before analysis. Consent was not obtained to use or publish individual level identified data from the participants and hence cannot be shared publicly. The de-identified data can be obtained from the corresponding author on reasonable request.

## List of abbreviations

ABS: Australian Bureau of Statistics
ACCHSs: Aboriginal Community Controlled Health Services
MBS: Medicare Benefits Schedule
GPs: General Practitioners
NT: Northern Territory
WA: Western Australia
PHC: Primary Health Care
AHPs: Aboriginal Health Practitioners
RANS: Remote Area Nurses
NTG: Northern Territory Government
HR: Human Resources
